# Prevalence of Primary Hypertension and Risk Factors in Grade XII Learners in KwaZulu-Natal, South Africa

**DOI:** 10.1155/2018/3848591

**Published:** 2018-07-02

**Authors:** Rajendra Bhimma, Elaene Naicker, Verena Gounden, Louansha Nandlal, Cathy Connolly, Sudesh Hariparshad

**Affiliations:** ^1^Department of Paediatrics and Child Health, Nelson R Mandela School of Medicine, School of Health Science, University of KwaZulu-Natal, Congella, Durban 4013, South Africa; ^2^Department of Chemical Pathology, University of KwaZulu-Natal and National Health Laboratory Services, Inkosi Albert Luthuli Central Hospital, Durban, South Africa; ^3^Department of Optics and Imaging, Nelson R Mandela School of Medicine, School of Health Science, University of KwaZulu-Natal, South Africa; ^4^Department of Biostatistics, School of Public Health, University of KwaZulu-Natal, South Africa; ^5^Department of Nephrology, Nelson R Mandela School of Medicine, School of Health Science, University of KwaZulu-Natal, Congella, Durban 4013, South Africa

## Abstract

Hypertension in childhood leads to hypertension in adult life, the strongest risk factor being obesity. This study determined the prevalence of primary hypertension and its risk factors in Grade XII learners in KwaZulu-Natal, South Africa, from March 2016 to June 2017. Weight, height, body mass index (BMI), random finger prick cholesterol and glucose, and spot urine for an albumin : creatinine ratio were measured. An average of three separate blood pressure readings taken was at least 5 minutes apart. Five hundred and sixty-four learners had weight, height, and BMI; 536 had random blood glucose; and 545 had cholesterol and random spot urine albumin : creatinine ratios measured. Prehypertension was detected in 168 (29.7%) and hypertension in 77 (13.7%) of learners. Ninety (15.9%) were overweight and 75 (13,3%) were obese. Hypercholesterolaemia was present in 58 (10.8%) and a high spot random urine albumin : creatinine ratio in 5 (1.0%). None had a high blood glucose level. Both prehypertension and hypertension in all learners showed a significant increase with increasing BMI. Six (1.0%) learners had metabolic syndrome. Female learners in other racial groups (defined as Indian, mixed race, and White learners), overweight, and obese learners showed significantly higher rates of hypercholesterolaemia. We showed overweight and obesity as risk factors for prehypertension and hypertension. This presages the need for an appropriate diet and adequate exercise in a child's school career.

## 1. Introduction

Hypertension has over the recent decades become a global health problem [1]. The criteria for hypertension in children and adolescents are complex as it entails correcting for age-, gender-, and height-specific reference values and as such pose a challenge in clinical practice [2]. Thus, both hypertension and prehypertension, though prevalent in childhood and increasing in frequency, remain widely underdiagnosed [3]. The global prevalence of hypertension in children and adolescents has been reported as 1 - 5% [4].

It is well established that hypertension in childhood and adolescence leads to hypertension in adult life and it is an independent risk factor for cardiovascular disease with other risk factors such as hypercholesterolaemia, hypertriglyceridaemia, low high-density lipoprotein cholesterol, truncal (central) obesity, hyperinsulinaemia, and metabolic syndrome [5, 6]. The strongest risk factor for paediatric hypertension is obesity; other factors include a family history of hypertension and male sex [7]. This association between excess weight and hypertension in childhood has been reported in several studies [8]. The prevalence of obesity has been exponentially increasing across both developed and developing countries [9–11]. Sorof* et al.* reported a three times higher prevalence of hypertension in obese compared to nonobese adolescents in a school-based hypertension and obesity screening study [8]. Among all demographic and clinical factors analysed, increasing body mass index (BMI) was the most strongly associated with hypertension.

According to Sorof and colleagues [12] the development of effective strategies to manage primary hypertension in children and adolescents requires an understanding of the condition in site-specific, community, or regional settings as these may have a bearing on modifiable factors such as dietary intake and activity levels. Children who are overweight, born preterm, or small for gestational age and of Black race are at increased risk for developing hypertension [13]. In the developed world, salt intake is generally above the recommended intake among children and a similar trend is seen in the developing world. Although a positive sodium balance is needed for growth in the first year of life, persistence of high salt intake subsequently can have deleterious cardiovascular consequences [14]. This is relevant since blood pressure in childhood tracks into adulthood and high salt intake is often associated with increased consumption of sugar-sweetened beverages (predisposing children to weight gain) [13].

In a previous study undertaken by Bhimma et al. in the central Phoenix Region of KwaZulu-Natal, South Africa in 2012 [15], the prevalence of prehypertension and hypertension among school-going children aged ≥6 to <18 years was estimated to be 2.1% (0.8–3.5%) and 2.6% (1.1–4.1%), respectively, similar to the 3-5% indicated in other reports in the literature globally [12, 15–19]. The risk of high blood pressure was found to rise with an increase in age, weight, height, waist circumference, increased triglyceride levels and heart rate, and family history of high blood pressure and diabetes, as well as suspected substance abuse [15]. A lack of exercise and an increased consumption of soft drinks were further associated with an increased risk for hypertension, which may be related to the increase in BMI associated with these lifestyle factors.

The study by Bhimma et al. was done in a single region (Phoenix) in KwaZulu-Natal in a wide age range of learners (≥6 to <18 years) and risk factors such as biochemical measurements of blood glucose, triglycerides, and high-density lipoprotein cholesterol levels were not reported. Phoenix is predominantly an Indian area and not truly representative of the other regions of KwaZulu-Natal [15]. In the present study, the racial demographics are more representative of the Province and we recruited only Grade XII learners to determine the cumulative effect of the risk factors for hypertension over the schooling years.

The aim of this study was to determine the prevalence of primary hypertension and associated risk factors in Grade XII learners in KwaZulu-Natal, South Africa, using a study sample more representative of the demographics of this Province. It is hoped that this study will help formulate strategies that will provide guidelines to regional health care policy makers on how to curb the imminent epidemic of increasing overweight and obesity and associated hypertension in learners.

## 2. Materials and Method

This was an observational, cross-sectional study on the prevalence of primary hypertension in Grade XII learners in 15 randomly selected schools from 5 districts in KwaZulu-Natal, South Africa, conducted from March 2016 to June 2017. Age, sex, race, and place of residence were recorded for each learner.

Certified trained nursing staff accompanied by a medically qualified doctor undertook the following biometric measurements: weight (kg) and height (cm). Weight measurements were undertaken with the learner wearing his/her school uniform but without a coat and other items that may increase weight, using a calibrated scale (Salter Salter Ultra Slim Bathroom Scale, Model SS3R®) with measurements in kilograms to one decimal space. Height measurements were done with the learner standing upright on a flat surface against a straight wall without shoes, heels together and the learners heels, buttocks, shoulders, and head touching the vertical wall surface with their line of straight sight aligned horizontally using a soft portable tape and measured to the nearest one centimetre. The tape measure was fixed to a straight wall and calibrated using a one metre ruler to ensure that the height measured using the tape fixed to the wall and the ruler were the same. Internal validation was done by one of the investigators measuring their height at the hospital using a stadiometer and comparing it to the height taken at the site. A single certified trained nurse at each site was allocated to do the height and weight measurements to avoid observer bias, although this could not be totally eliminated between sites. BMI was calculated as weight/height^2^ (kg/m^2^) and categorized according to the Center for Disease Control and Prevention (CDC) age- and sex-specific growth charts [20]. Learners were classified as normal if the BMI was ≤85th percentile (BMI ≥ 18.5 kg/m^2^ and <25 kg/m^2^) for age and sex, overweight if the BMI was ≥85th percentile but <95th percentile (BMI ≥ 25 kg/m^2^ and <30 kg/m^2^), and obese if the BMI was ≥95th percentile (BMI ≥ 30 kg/m^2^ and <35 kg/m^2^).

All learners also had the following clinical and laboratory assessments done: blood pressure measurements (mmHg), urinary dipsticks analysis, random blood sugar (glucometer reading), random cholesterol, and a random urine albumin: creatinine ratio on a spot urine sample.

Blood pressure measurements were done using an appropriate size cuff with the child at rest and seated upright. The right arm was maintained in a relaxed position and supported with the cubital fossa at the level of the heart. An average of three separate readings taken at least 5 minutes apart was recorded and the definition of hypertension used in this study was based on the Fourth Report on the Diagnosis, Evaluation, and Treatment of High Blood Pressure in Children and Adolescents [21] which states that sustained systolic or diastolic blood pressure measurements should be in the 95th percentile for a respondent to be classified as hypertensive [22]. Systolic and diastolic blood pressures were measured using the oscillometric method, employing an automated blood pressure monitor Edan Model 3® done by the same individual to eliminate operator bias. Recommendations for cuff sizes published by the National High Blood Pressure Education Working Group on Hypertension control in Children and Adolescents (1996) were followed. The width: length ratio of the inflatable bladder used was at least 1:2 with width covering a minimum of 40% of the arm at a point midway between the olecranon and acromion and length covering 80-100% of the circumference of the arm.

Hypertension is defined by the Forth Report on the Diagnosis, Evaluation, and Treatment of High Blood Pressure in Children and Adolescents which classifies BP as follows: normal if <90th percentile; prehypertension if ≥90th to <95th percentile or >120/80 mmHg in adolescents; Stage 1 HPT if between ≥95th to 99th percentile plus 5 mmHg; Stage 2 HPT if >99th percentile plus 5 mmHg [21].

Biochemical measurements of random blood glucose and random cholesterol levels were made by fingerpick testing using the Accutrend Plus Cobas® machine (Roche Diagnostics®, Germany). Hyperglycaemia was defined as a random glucometer reading >11.1 mmol/l of blood glucose and hypercholesterolaemia as a random cholesterol level ≥4.6 mmol/l [23].

A fresh spot random clean-catch midstream sample of urine collected in a sterile urine collector was used for measurement of albumin: creatinine ratio and urinary dipsticks analysis. Urinary dipsticks analysis was done using Combur 9 Test® dipsticks (Cobas, Roche Diagnostics®) according to the manufacture's specifications. Measurement for albumin: creatinine ratio was undertaken at the National Health Laboratory Services on the Advia 1800 Chemistry Analyser® (Siemens, Tarrytown, NY) at the Inkosi Albert Luthuli Central Hospital. For males a random spot urine albumin: creatinine ratio in the range 2.5-25 mg/mmol was defined as normal. Greater than this range was considered as frank proteinuria. For females, the normal range was 3.5-35 mg/mmol and greater than this range was defined as frank proteinuria. Metabolic syndrome was defined by three metabolic abnormalities including three of the following: obesity, hypertension, high random cholesterol, or hyperglycaemia [23].

The measurements were all done at the schools using a suitable venue that was allocated by the school Principal. The basic science students were involved in setting up and assisting the students with the completion of the questionnaires. All equipment and staff were transported to the sites. All measurements were made by a single observer at each site to eliminate interobserver error. At the time of data collection if a value for any of the parameters measured was abnormal, the staff alerted the doctor on site who gave the learner a referral letter to a health care centre stating the problem. This was done following a private discussion with the student and with their full consent. Learners diagnosed with hypertension, hypercholesterolaemia, or high blood sugar were referred to the nearest health care centre for further evaluation and appropriate treatment. Overweight and obese learners with normotension were referred to the dietetics department of their local hospitals or Inkosi Albert Luthuli Central Hospital. Inclusion criteria for the study were all available Grade XII learners from randomly selected schools in KwaZulu-Natal whose parents gave written informed consent and who assented to participate in the study. Exclusion criteria were failure to obtain consent and/or assent to participate in the study, learners with known chronic diseases such as chronic kidney disease, heart disease, and diabetes that were likely to be a known secondary cause of hypertension.

### 2.1. Data Collection

The study team is comprised of nurses and doctors from the Nephrology Units (adult and paediatric) at the Inkosi Albert Luthuli Central Hospital who volunteered to undertake the study as part of their commitment to the World Kidney Day (Theme: “Kidney Disease & Children. Act Early to Prevent It!”) commemorated on the 10 March 2016. In addition, medically trained staff (nurses and doctors) working in peripheral hospitals, private hospital in the Durban Functional Region and basic science students undertaking their Masters in Science degree from the University of KwaZulu-Natal, School of Laboratory Medicine, volunteered their services. All data were collected on a data sheet and given a case number. No identifiers were used so that the data was totally anonymous. Information was captured on an Excel password protected spread sheet using Microsoft Office version 10® and transported into an SPSS® statistical package for further statistical analysis.

### 2.2. Statistical Analysis

#### 2.2.1. Sample Size

The requisite sample size was determined using an estimation of population size in the area, with a minimum representative sample taken from each school. A sample size of 316 was required to estimate the level of hypertension in school children to within 5% with a probability of 95% and assuming an estimate of 10% hypertension in the population. Since schools were the sampling unit, a design factor of 1.5 was used to adjust for the correlation within schools which increased the sample size to 474. The sample size was also increased by 15% to account for incomplete data and consent refusal. Thus, the total sample size required for this study was 560 children.

Statistical analysis was performed using the IBM SPSS Statistics® software package. Histograms and box-whisker plots were used to visually assess data distributions and check for outliers. The Kolmogorov-Smirnov test was used to assess whether the distributions differ from normal distribution. Nonparametric statistics was used to describe and test associations for variables with significantly skewed data, whereas parametric and summary statistics were used for variables that conform to normal distribution. Comparisons were made between hypertensive and nonhypertensive groups using the 2 *χ*^2^ test in case of categorical variables. Continuous variables were summarized as a mean ± standard deviation (SD), and comparisons among the groups were based on the Student t-test. Differences were considered statistically significant when p < 0.05. Statistical differences were also sought for blood pressure in subjects who were overweight or obese versus those that were normal.

#### 2.2.2. Ethical Considerations

Permission to conduct this study was obtained from the Provincial Department of Health and Provincial Department of Basic Education. The school Principal was informed and permission obtained prior to study commencement to undertake the study on school premises. Ethical approval to conduct the study was obtained from the Biomedical Research Ethics Committee of the University of KwaZulu-Natal (BE032/16). Written informed consent was obtained from parents or legal guardians. This was achieved by learners at each site being briefed at least two weeks prior to data collection with a study information sheet and consent forms attached given to learners to hand over to their parents or legal guardian. In addition, all learners had to have a signed assent form before participation in the study.

## 3. Results

**Figure 1:**
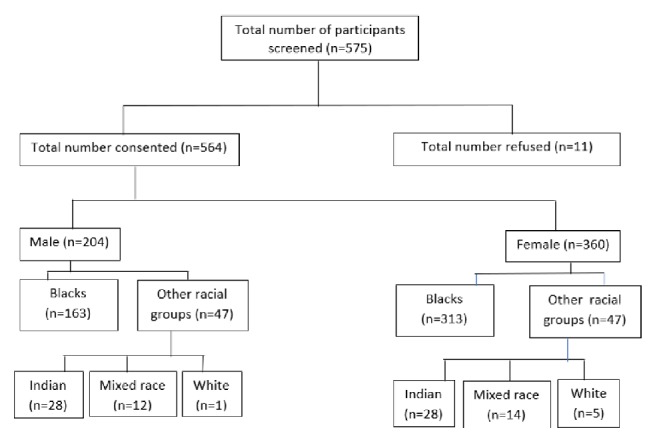
Study population of Grade XII learners from 15 schools in 5 different districts in KwaZulu-Natal.

A total of 575 learners were willing to participate and present at school on the day of the study. All learners had a detailed history taken by the medical officer on site to exclude any chronic diseases that may result in the development of secondary hypertension. Eleven parents refused consent and these learners were excluded. Complete data for height, weight, BMI, and blood pressure was obtained in 564 learners that were included in the study (Figure 1). Random glucose and cholesterol were measured in 536 learners as 28 refused to have finger prick bloods done and spot urinary albumin: creatinine ratios were measured in 545 learners as the urine from 19 learners unfortunately leaked in transit to the laboratory. All learners were of South African nationality, White. Given the small numbers of learners in each of the racial groups other than Black Africans, Indian, mixed race, and White learners were grouped together as other race groups giving a total of 88 (15,6%) learners in this collective group (Figure 1). The mean age of learners was 17.8 ± 1.1 years with a range of 16.2 – 21.7 years.

The mean height of the learners was 1.72 ± 0.12 metres (range 1.62-1.83) for males and 1.61 ± 0.22 metres (range 1.52 – 1.74). One hundred and thirty-one (23.2%) learners were <5th percentile for age-for-height based on the Center for Disease Control and Prevention (CDC) age-and-sex specific growth charts [20].

Prehypertension was detected in 168 (29.7%) and hypertension in 77 (13.7%) of learners. The rates of elevated blood pressure (prehypertension and hypertension combined) in males and females were 52.5% and 38.3%, respectively. Males were more significantly likely to be prehypertensive compared to females (35.8% versus 26.4%, OR: 1.8, 95%CI: 1.2-2.6, p < 0.004), and also more likely to be hypertensive (16.7% versus 11.9%, OR: 1.8, 95% CI: 1.1-3.3, p < 0.02) (Table 1). On comparing Black African learners to other racial groups, there was no statistically significant difference with prehypertension in both groups. However, learners in the other racial groups were more likely to be hypertensive compared to Black African learners (22.7% versus 12.9%, OR: 2.2 95% CI: 1.2-4.0, p < 0.01). Thus, hypertension was significantly associated with race and sex (Table 1). None of the learners had a history of taking antihypertensive medication or had a previous diagnosis of hypertension.

The mean weight was 61.62 ± 14.64 kilograms (range 32.14-140.32). Fifty-four (9.6%) of learners were <5th percentile and 61 (10.8%) were >95 percentile for age-and sex-specific weight. Three-hundred and ninety-nine (70.7%) had a normal BMI, 90 (15.9%) were overweight, and 75 (13.3%) were classified as obese. None had a BMI >35 kg/m^2^. Females were more likely to be overweight than males (OR: 1.4, 95%CI: 1.9-2.3, p < 0.02) and also more likely to be obese (OR: 1.8, 95% CI: 1.0-3.1, p < 0.04). On comparing Black African learners to those from other racial groups, the latter group was more likely to be overweight (OR: 2.2, 95% CI: 1.3-3.9, p < 0.004); however there were similar rates of obesity in both groups (OR: 1.2, CI 0.6-2.5, p < 0.5) (Table 1).

Both prehypertension and hypertension in all learners were associated with significantly higher BMI (Table 1). Prehypertension was 26.3% in learners with a normal BMI; 35.6% in those that were overweight; and 41.3% in obese learners (OR: 1.8, 95% CI 1.1-.30, p < 0.02 and OR: 3.7, 95% CI: 2.0-6.9, p < 0.001), respectively. Hypertension was 9.5% in learners with normal BMI; 16.7% in learners that were overweight; and 32.0% in obese learners (OR: 2.4, 95%CI: 1.2-4.6, p < 0.01 and OR: 8.1, 95% CI: 4.1-16.0, p < 0.001), respectively.

When stratified for race, there was no association of prehypertension or hypertension in Black African learners who were overweight when compared to those with normal BMI (OR: 1.6, 95% CI: 0.7-3.7, p > 0.07). However, both prehypertension and hypertension in Black African learners were significantly associated with obesity (OR: 4.7, 95% CI: 2.4-9.0, p < 0.001; OR: 7.4, 95% CI: 2.4-9.0, p < 0.001), respectively (Table 2). In learners in other racial groups, only hypertension was significantly associated with overweight (OR: 4.7, 95% CI 1.2-17.8, p < 0.02) and obesity (OR: 9.3, 95% CI: 2.1-42.1, p < 0.004). However, given the small numbers of learners in this group, the results must be treated with reserve. When hypertension and BMI were stratified by sex, both hypertension and hypertension were associated with overweight and obesity in females. However, in males only hypertension was associated with overweight and obesity (Table 2).

Hypercholesterolaemia was present in 58 (10.8%) of 536 learners. Thirty-four (6.3%) of learners with hypercholesterolaemia had normal blood pressure, 11 (2.1%) prehypertension, and 13 (2.4%) hypertension. Female learners in the other racial groups and overweight and obese learners showed an association with hypercholesterolaemia (Table 3). Only 6 (1.0%) of the 559 learners had all necessary criteria for the metabolic syndrome. There was no significant association when adjusted for race or sex.

Only 5 (1.0%) of 545 learners tested had a spot random urine albumin: creatinine ratio that was high. There was no significant association between learners with a high albumin: creatinine ratio and prehypertension or hypertension, sex, race, or BMI. Also, none of the learners had an abnormal blood glucose level.

Hypercholesterolaemia, random blood glucose, and spot urine albumin: creatinine ratios showed no signs of associated risk with hypertension.

## 4. Discussion

In our study, we found a high rate of prehypertension (29.7%) and hypertension (13.7%) in Grade XII learners in KwaZulu-Natal, South Africa. This is much higher than a report in a previous study undertaken by Bhimma et al. in the central Phoenix Region of KwaZulu-Natal, South Africa, in 2012; the prevalence of prehypertension and hypertension among school-going children aged ≥6 to <18 years was estimated to be 2.1% and 2.6%, respectively [15]. However in a study undertaken in grades 11 and 12 (i.e., pre- and matriculation years) in 10 schools (nine public schools and one private school) in nine provinces in South Africa over a ±9–10-year period, with more than 96% of learners being Black African, and mostly from penurious backgrounds, hypertension was reported in 12% of females and 16% of males [24], very similar to the findings in our study. Rates of hypertension in sub-Saharan Africa have been noted to be increasing among adults [25–27].

Detection of hypertension in children is important as it has been shown to have potentially severe consequences such as target organ damage including left ventricular hypertrophy, kidney damage and progression of chronic kidney disease, accelerated pathological vascular changes, and subtle neurological effects like reduced cognitive function [28, 29]. Whilst no long-term cohort studiers have directly linked hypertension in childhood to cardiovascular events in adults, it is well known that hypertension in childhood and adolescents tracks hypertension into adulthood [30].

Our study also found 15.9% of Grade XII learners to be overweight and 13.3% to be obese with females and learners from other racial groups being more likely to be overweight or obese. These findings are in line with other data from South Africa [31–33]. Previous studies have shown that the strongest risk factor for hypertension in childhood is obesity, male sex, and a family history of hypertension [7, 34]. These findings are supported by our study in which we showed overweight and obese learners to be more likely to be both prehypertensive and hypertensive compared to learners with normal weights as determined by BMI. Also, we showed that males were more likely to be both prehypertensive and hypertensive compared to female learners.

In adults, the rising rate of hypertension in sub-Saharan Africa has been associated with the rising rates of obesity, particularly in urban areas [10, 11]. These high rates of obesity-associated hypertension are probably due to the epidemiological transition to a more Westernised lifestyle with increased television viewing, Internet surfing and smart phones games, and other applications, leading to a more sedentary lifestyle, as well as dietary habits consisting of mainly processed foods. Indeed, a high consumption of energy-dense diets and a sedentary lifestyle account for the increasing burden of overweight and obesity in urban settings and now have also infiltrated into less developed areas [35]. There is substantial evidence that the risk of noncommunicable diseases such as coronary heart disease, ischaemic stroke, and type 2 diabetes mellitus increases steadily with increasing BMI [36].

The increasing prevalence of obesity is projected to increase worldwide to 50% and 60% by 2030 and 2050, respectively [37]. Although rates of hypertension are less clear, increasing BMI has been consistently associated with rising blood pressure throughout childhood [38–40]. This strong association of obesity-associated hypertension in less developed countries portends that these nations face similar difficulties to developed nations in overcoming lifestyle choices in combating obesity and hypertension.

In our study, there was no association of race on the rates of prehypertension in Grade XII learners. However, learners classified as belonging to other racial groups were more likely to be hypertensive compared to Black African learners (22.7% versus 12.0%, p < 0.01). This high risk for hypertension among minority groups (excluding Asians) has been demonstrated in several other studies [18, 41–43]. Our study had very few White learners and no Hispanic learners.

To help formulate strategies that will provide guidelines to regional health care policy makers submissions will also be made to the Department of Education and Department of Health to advise health care teams to do routine health screening in all learners, including blood pressure measurements. In addition, to sensitize health care workers to this looming epidemic, the data will be presented at local and international congresses, placed on hospital websites, as well as submitted to the lay press.

## 5. Study Limitations

This study has some limitations. Firstly, the criteria for the definition of hypertension in children are arbitrary and to a certain extent artificial. We used normative data from blood pressure in children and adolescents based on the Fourth Report on the Diagnosis, Evaluation, and Treatment of High Blood Pressure in Children and Adolescents [21]. Whether these normative data are applicable to children and adolescents in other parts of the world or to different ethnic backgrounds is unknown. Secondly, the numbers of Grade XII learners in the category other racial groups were relatively small (88 learners) and therefore comparisons may not have been sufficiently powered to show significant differences. Thirdly, in common with other studies [11, 44, 45], we used the average of three blood pressure measurements at a single visit taken at least five minutes apart with the leaner at rest. Ideally for the diagnosis of hypertension, blood pressure should be measured on at least three separate occasions with sustained systolic or diastolic blood pressure measurements above the 95th percentile for a respondent to be classified as hypertensive [21]. It is possible that the prevalence of hypertension would have been lower where these criteria were used to determine hypertension [46]. Fourthly we used BMI as the sole measure of obesity as in other reports [11, 12, 47, 48]. For more specific assessment of fat mass, other measurements estimating adiposity such as waist circumference, bioelectrical impedance, and MRI can be used. However for population-based studies, BMI correlates well with these other measures and remains widely used as a definition of obesity in hypertension research [11, 12, 47, 48]. Fifthly we were not able to accurately document levels of physical activity and substance abuse and consumption of processed food, all of which are variables indicative of hypertensive risk. Sixthly we could not accurately document the role of family history in our learners as many were unsure of the parents' blood pressure status and in many instances, parents failed to disclose their illnesses on the questionnaire form.

## 6. Conclusion

Our study showed a high prevalence of overweight and obesity as risk factors to prehypertension and hypertension in Grade XII learners in KwaZulu-Natal. To overcome this health care professionals, health authorities, and governments will need to educate learners on major lifestyle changes including* inter alia* improved dietary habits, doing more exercise with increased participation in sports, and encouraging to desist from smoking and others such unsavoury habits.

## Figures and Tables

**Table 1:** Stratification of hypertension by gender, race, and body mass index.

	Normotensive	Prehypertensive				Hypertensive			
	n (%)	n (%)	OR	95% CI	p value	n (%)	OR	95% CI	p value
**Total**	319 (56.6)	168 (29.8)				77 (13.6)			
**Gender**									
** Male**	97 (47.6)	73 (35.8)	1,8	(1.2 - 2.6)	0,004	34 (16.7)	1,8	(1.1 - 3.0)	0,02
** Female**	222 (61.7)	95 (26.4)				43 (11.9)			
**Race**									
** Black**	275 (57.8)	144 (30.2)				57 (12.0)			
** Other racial groups**	44 (50.0)	24 (27.3)	1,0	(0.6 - 1.8)	0,9	20 (22.7)	2,2	(1.2 - 4.0)	0,01
**BMI**									
** Normal**	256 (64.2)	105 (26.3)				38 (9.5)			
** Overweight**	43 (47.8)	32 (35.6)	1,8	(1.1-3.0)	0,02	15 (16.7)	2,4	(1.2-4.6)	0,01
** Obese**	20 (26.7)	31 (41.3)	3,8	(2.1-6.9)	<0.001	24 (32.0)	8,0	(4.1-16.0)	<0.001

BMI: body mass index.

Other racial groups: comprised of South African, Indian, White, and mixed race.

OR: odds ratio.

CI: confidence interval.

**Table 2:** Stratification of hypertension by body mass index, sex, and race.

		Normotensive	Prehypertensive				Hypertensive			
		n (%)	n (%)	OR	95% CI	p value	n (%)	OR	95% CI	p value
**Black African** ^**1**^	**BMI**	275 (57.8)	144 (30.3)				57 (12.0)			
	Normal	224 (64.7)	90 (26.0)				32 (9.3)			
	Overweight	35 (52.2)	24 (35.8)	1.7	1.0-3.0	0.07	8 (11.9)	1.6	0.7-3.7	0.3
	Obese	16 (25.4)	30 (47.6)	4.7	2.4-9.0	<0.001	17 (27.0)	7.4	3.4-16.2	<0.001

**Males**	**BMI**	97 (47.6)	73 (35.8)				34 (16.7)			
	Normal	81 (51.9)	56 (35.9)				19 (12.2)			
	Overweight	10 (35.7)	11 (39.3)	1.6	0.6-4.0	0.3	7 (25.0)	3.0	1.0-8.9	0.049
	Obese	6 (30.0)	6 (60.0)	1.4	0.4-4.7	0.5	8 (40.0)	5.7	1.8-18.3	0.004

**Females**	**BMI**	222 (61.7)	95 (26.4)				43 (11.9)			
	Normal	175 (72.0)	49 (20.2)				19 (7.8)			
	Overweight	33 (53.2)	21 (33.9)	2.3	1.2-4.3	0	8 (12.9)	2.2	0.9-5.5	0.02
	Obese	14 (25.5)	25 (45.5)	6.4	3.1-13.2	<0.001	16 (29.1)	10.5	4.5-24.9	<0.001

OR: odds ratio.

CI: confidence interval.

^1^Other racial groups not included because of the small sample size.

**Table 3:** Stratification of cholesterol by hypertension, sex, race, and BMI.

	Cholesterol (n = 536) (missing = 28)					
	Normal (<4.5)		Abnormal (>=4.5)		Total	*p* value
	n	%	n	%	n	
**Cholesterol**	478	89%	58	11%	536	
**Hypertension**						
Both normal	269	89%	34	11%	303	0,06
Prehypertensive	146	93%	11	7%	157	
Hypertensive	63	83%	13	17%	76	
**Sex**						
Male	186	93%	13	7%	199	0,01
Female	292	87%	45	13%	337	
**Race**						
African	414	92%	34	8%	448	<0.001
Other racial groups	64	73%	24	27%	88	
**BMI**						
Normal	348	93%	28	7%	376	< 0.001
Overweight	73	83%	15	17%	88	
Obese	57	79%	15	21%	72	

Other racial groups: comprised of South African, Indian, White, and mixed race.

## Data Availability

The data used to support the findings of this study are available from the corresponding author upon request.
